# Peptide hormone sensors using human hormone receptor-carrying nanovesicles and graphene FETs

**DOI:** 10.1038/s41598-019-57339-1

**Published:** 2020-01-15

**Authors:** Sae Ryun Ahn, Ji Hyun An, Seung Hwan Lee, Hyun Seok Song, Jyongsik Jang, Tai Hyun Park

**Affiliations:** 1 School of Chemical and Biological Engineering, Seoul National University, Seoul, 08826 Republic of Korea; 2 Semiconductor R&D Center, Samsung Electronics, Hwaseong, Gyeonggi 18448 Republic of Korea; 3 Department of Bionano Engineering, Hanyang University, Ansan, 15588 Republic of Korea; 4 Sensor System Research Center, Korea Institute of Science and Technology, Seoul, 02792 Republic of Korea

**Keywords:** Antisense elements, Blood urea nitrogen

## Abstract

Hormones within very low levels regulate and control the activity of specific cells and organs of the human body. Hormone imbalance can cause many diseases. Therefore, hormone detection tools have been developed, particularly over the last decade. Peptide hormones have a short half-life, so it is important to detect them within a short time. In this study, we report two types of peptide hormone sensors using human hormone receptor-carrying nanovesicles and graphene field-effect transistors (FETs). Parathyroid hormone (PTH) and glucagon (GCG) are peptide hormones present in human blood that act as ligands to G protein-coupled receptors (GPCRs). In this paper, the parathyroid hormone receptor (PTHR) and the glucagon receptor (GCGR) were expressed in human embryonic kidney-293 (HEK-293) cells, and were constructed as nanovesicles carrying the respective receptors. They were then immobilized onto graphene-based FETs. The two hormone sensors developed were able to detect each target hormone with high sensitivity (ca. 100 fM of PTH and 1 pM of GCG). Also, the sensors accurately recognized target hormones among different types of peptide hormones. In the development of hormone detection tools, this approach, using human hormone receptor-carrying nanovesicles and graphene FETs, offers the possibility of detecting very low concentrations of hormones in real-time.

## Introduction

Hormones in very low concentration control and regulate the activity of certain cells and organs in the human body^1^. Numerous diseases, such as osteoporosis, cardiovascular, adenoma, hyperplasia, and cancer, are related to hormone imbalance^2–7^. Therefore, significant efforts have been made over the last decade in the development of tools to detect hormones. Well-established detection methods for hormone detection are normally based on immunometric assays^8–10^. However, they have the limitations of selectivity, sensitivity, and time-consuming problems for diagnosis and health screening. Peptide hormones have a short half-life, so in order to detect them, they must be detected within a short time after taking them from the body^11^. In the case of hormones with short half-life, real-time detectable sensors are needed.

Parathyroid hormone (PTH) is also called parathormone, and secreted in parathyroid glands. This peptide hormone plays an important role in the regulation of bone and mineral metabolism. It is secreted when the concentration of calcium in the blood is low, causing the calcium to dissolve into the blood from the osteoclasts. It is known that adenoma, hyperplasia, and cancer cause high concentration of PTH in blood. PTH in the human body acts as a ligand to the parathyroid hormone receptor (PTHR), a type of GPCR. PTH is an 84-amino acid peptide hormone, and its half-life is (2–4) min^12^. PTH has been measured by RadioImmunoAssay (RIA) and Enzyme-Linked ImmunoasSAy (ELISA)^13–15^. Recently, the most common method used is ELISA, which has a detection limit of 3 pM. However, this methods require pretreatment and reaction with reagents. Moreover, because PTH exists in various fragments in the blood, it is necessary to measure it using different antibodies for each fragment. As the degree of response *in vivo* varies depending on the site or length of the fragments, it is necessary to develop a method of measuring that is similar to a real biological system. Glucagon (GCG) is also a peptide hormone produced in the alpha cells of the pancreas, and also acts as a ligand to the glucagon receptor (GCGR). This hormone regulates the concentration of glucose in the bloodstream, as opposed to insulin^16^. When the glucose level falls too low, glucagon is secreted from the pancreas to convert glycogen into glucose. This hormone is also related to pancreatic tumors. The tumors can cause abnormally high glucagon level. GCG is a 28-amino-acid peptide hormone, and its half-life is (3–6) min. GCG has also been measured by RIA and ELISA (limit of detection: 3 nM) methods. This method also has the same limitations as the above-mentioned PTH measurement method. Therefore, the key factor is detecting peptide hormones from the body with high selectivity, and as soon as possible.

The GPCRs can recognize target substances in the body. Therefore, the GPCR can be applied to a sensor that can detect a target substance with high selectivity^17–20^. In our previous research, FET biosensors using human GPCRs have demonstrated the ability to successfully identify target substances^21–30^. In particular, sensors using human PTHR has enabled biosystem-like measurements by identifying different signals for different peptide hormone fragments^28^. GPCR-carrying nanovesicle-based FET sensors showed high performance real-time detection^22,31,32^. They were able to detect target substances with high sensitivity and selectivity. Also, usage of FET sensors showed highly rapid response (on a time scale of less than 1s). Among transducer materials, graphene has been useful, because it can provide excellent electrical and optical properties^33^. In addition, graphene provides excellent sensitivity, stability and a rapid response^34–37^. Previous study has shown that biosensor using graphene-FET and human GPCR-carrying nanovesicles exhibit excellent sensor characteristics^38^. Here, two kinds of hormone sensors were successfully demonstrated to detect PTH and GCG using graphene FET sensors fabricated with PTHR- and GCGR-carrying nanovesicles. PTHR and GCGR were expressed in HEK-293 cells, and then nanovesicles were produced. Each of these receptor-carrying nanovesicles was then immobilized on a graphene-based FET sensor, and constructed with one or other of the two hormone sensors. These sensors can be developed as a tool that can enable diagnosis by detecting hormones. They will also be useful as screening tools to discover alternative molecules.

## Results

### Construction of human hormone receptor-carrying nanovesicles and functional characterization of hormone receptors

**Figure 1:**
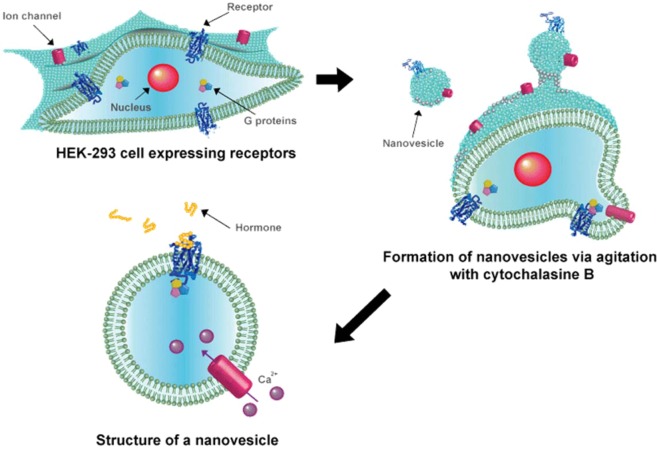
Schematic of the construction of human hormone receptors-carrying nanovesicles.

HEK-293 cells were transfected with pDsRed-N1 containing human PTHR and GCGR, and then stable cell lines were constructed. After, the hormone receptor-carrying nanovesicles were constructed as shown in Fig. 1. Detailed processes can be found in our previous studies^31,38,39^.

**Figure 2:**
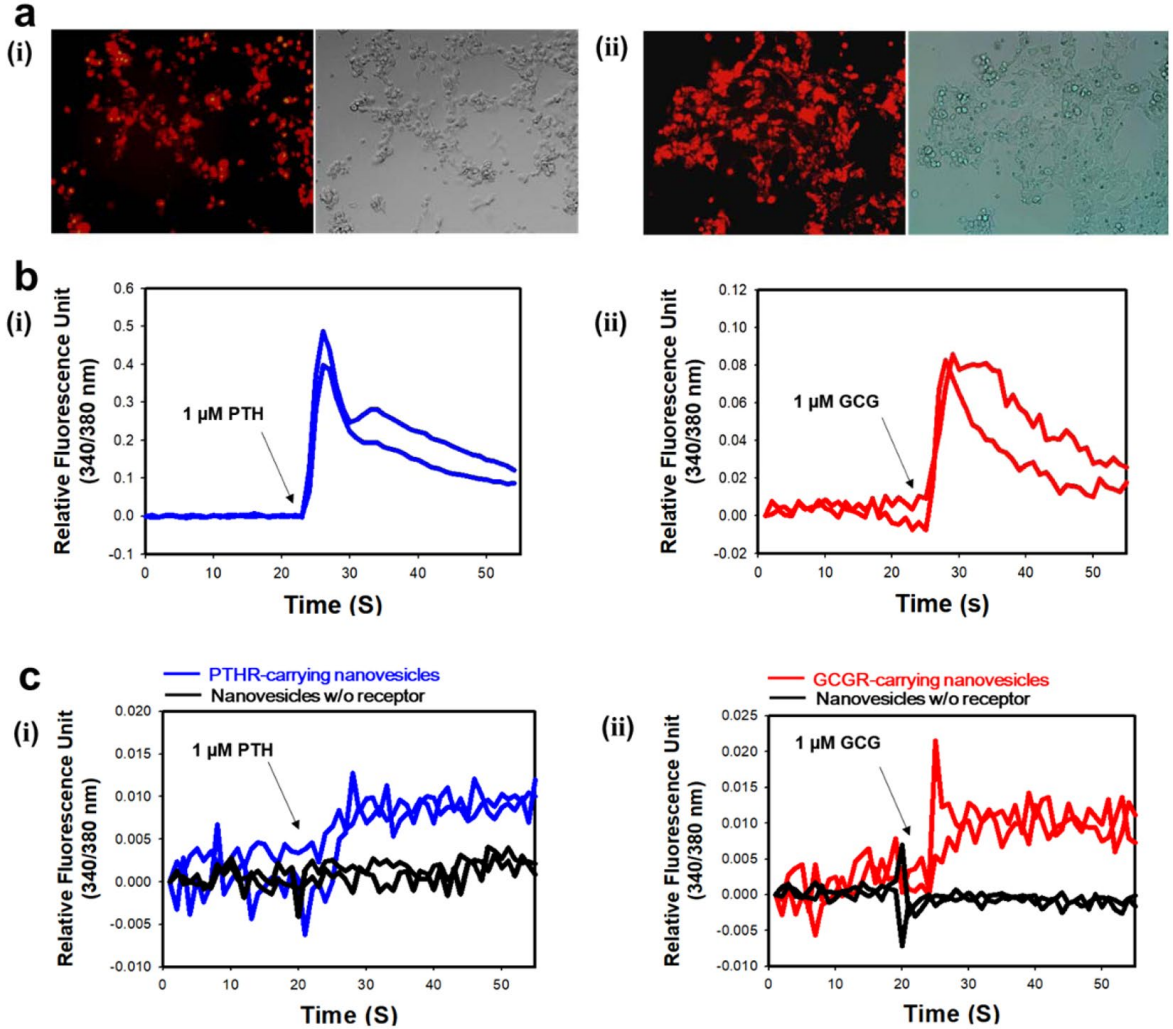
(**a**) Fluorescence images (left) and bright field images (right) of (i) PTHR-expressing cells, and (ii) GCGR-expressing cells. (**b**) Calcium signal analysis of (i) PTHR-expressing cells upon the addition of 1 µM of PTH, and (ii) GCGR-expressing cells upon the addition of 1 µM of GCG. (**c**) Calcium signal analysis using (i) PTHR-carrying nanovesicles upon the addition of 1 µM of PTH, and (ii) GCGR-carrying nanovesicles upon the addition of 1 µM of GCG.

Since PTHR and GCGR genes are inserted in the pDsRed N-1 vector, expression in HEK-293 cells can be easily confirmed by fluorescence microscopy. Figure 2a shows the expression of the hormone proteins tagged with the DsRed protein as a fluorescence image. Figure 2a(i) shows that PTHR with DsRed exhibits red fluorescence and was well expressed in cells. Figure 2a(ii) also shows the appearance of GCGR with DsRed can be confirmed by red fluorescence. The left images are fluorescence microscope images, and the right images are cell images obtained by applying the bright field. Comparison of fluorescence images with bright field images indicate that the expression rate is 80–90%. Calcium signal analysis was performed to confirm the function of these expressed hormone receptors (Fig. 2b). Figure 2b(i) shows that the relative fluorescence unit (340/380 nm) increases with increasing intracellular calcium concentration when stimulated with 1 μM PTH to cells expressing PTHR. Figure 2b(ii) also shows that the signals are increased when GCGR-expressed cells were stimulated with 1 μM GCG. These results confirmed that the two receptors, GPCRs along the cAMP signaling pathway, are stimulated by target hormones to generate intracellular signals which cause intracellular calcium influx. Also, this indicates that the hormone receptor-expressing cells with the accessory protein DsRed are able to carry out intracellular signaling normally.

Then stable cell lines were produced to increase the number of receptors present on the nanoveislce. The hormone receptor cells were treated with cytochalasin B, which destabilizes the cell membrane and makes the cell membrane unstable, and then gently agitated. Hormone receptor-carrying nanovesicles were produced by budded off from receptor-expressing HEK-293 cells. The constructed nanovesicles have not only receptors but also G proteins and other intracellular signalling machineries. To investigate whether the produced nanovesicles carrying hormone receptors are stimulated by the respective hormones and have intracellular signal transduction into the nanovesicles, calcium signal analysis was performed. Figure 2c(i) shows that the PTHR-carrying nanovesicles exhibit signals when stimulated with 1 μM PTH (blue line), compared to nanovesicles made with HEK-293 that are not expressed at all (black line). Figure 2c(ii) also shows the same content as the calcium signals for the GCGR-carrying nanovesicles (red line). The red line pick to 0.02 at around 25 seconds, seems to be an artifact caused by injection of GCG, an effect often observed in experiments. The actual signal is correct to see 0.01 except for artifacts. These results indicate that the nanovesicles maintain the intrinsic function of hormone receptor cell signaling. This indicates that the change in signal is due to a calcium influx from outside of the cell or nanovesicle. However, the calcium signal does not fully recover to baseline in the case of nanovesicles. This is perhaps due to a lack of ATP, ion pumps and calmodulin that may prevent nanovesicles from discharging calcium ion^22,40,41^.

### Fabrication and characterization of hormone sensors

**Figure 3:**
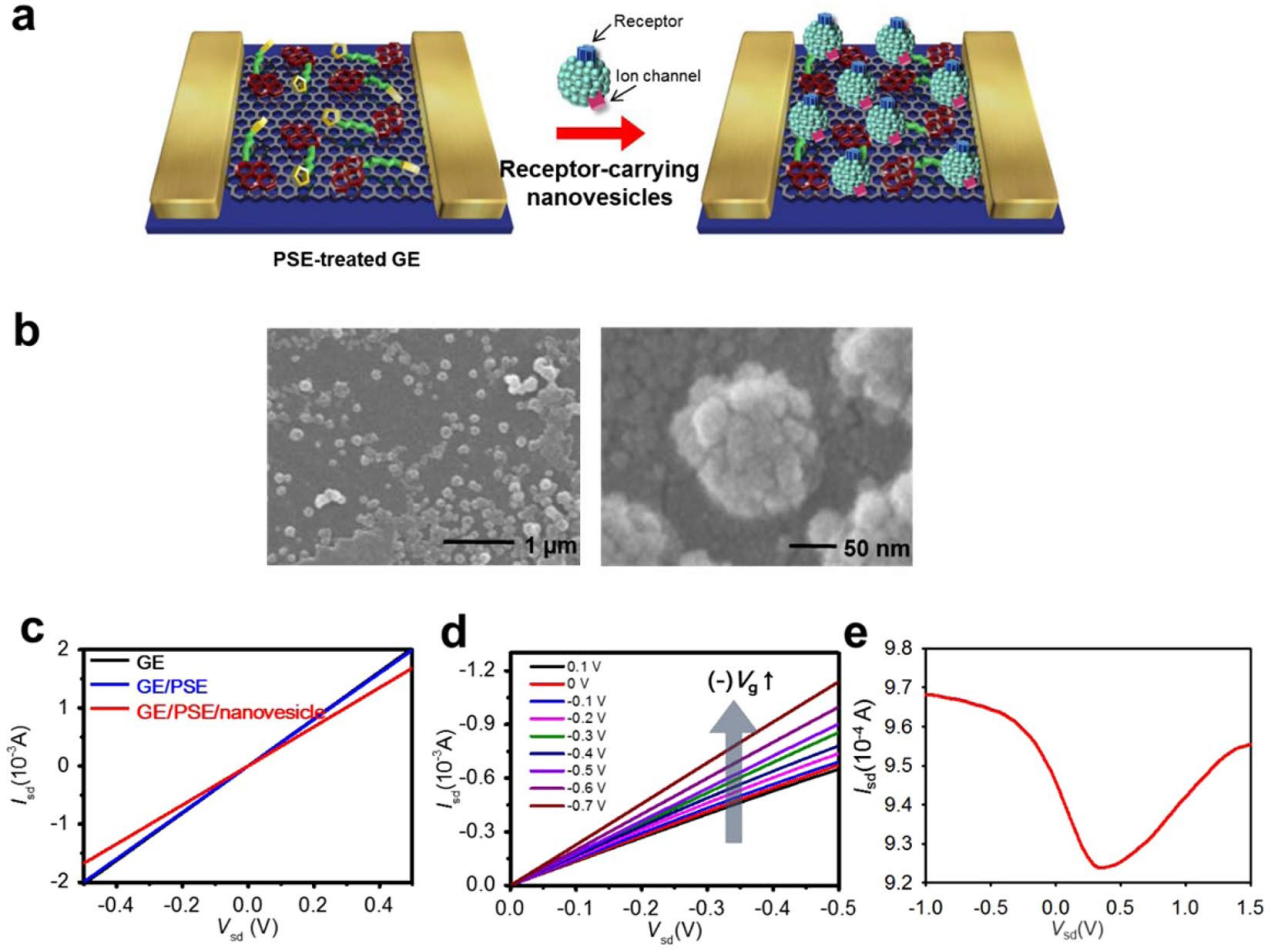
Fabrication and characterization of hormone sensors. (**a**) Schematic of the fabrication process. (**b**) FE-SEM image of a GE channel after the immobilization of nanovesicles. (**c**) Current–voltage (*I–V*) curves of the hormone sensor before and after the immobilization of nanovesicles. (d) Output characteristics of a FET-type hormone sensor (*V*_g_: (0.1 to –0.7) V in –0.1 V steps, and *V*_sd_: (0 to –0.5) V in –50 mV steps). (**e**) Transfer characteristics of the FET-graphene sensor for a constant *V*_sd_ = −100 mV in *V*_g_, –1 to 1.5 V.

Figure 3a shows a schematic of the immobilization of the nanovesicles on graphene electrode (GE). Based on our previous research^38^, the CVD-grown graphene was in the form of a single layer. Therefore, the thickness of the graphene channel was estimated to be about 0.8 nm, which is the thickness of a single layer of graphene. The length and width of the graphene pattern was about 50 µm × 50 µm and this graphene pattern was utilized as the channel region. Then, proteins of the nanovesicles interacted with the succinimidyl groups of the PSE as the linker molecules, forming stable peptide bonding. To remove excess PSE, the GEs were washed three times with pure methanol before immobilization of the nanovesicles. To visualize the immobilization of the nanovesicles on the graphene surface, field-emission scanning electron microscopy (FE-SEM) measurement was carried out. Figure 3b shows the FE-SEM image of the nanovesicles immobilized on graphene. This shows that the diameter of the nanovesicles produced is about (100 to 150) nm. In order to utilize the electrical characteristics of the nanovesicle-immobilized graphene-FET configuration, current–voltage (*I–V*) measurements were performed (Fig. 3c). Figure 3c shows the changes in the *I–V* values before and after PSE treatment, and nanovesicles immobilization on the surface of the GE channel. Although the *dI/dV* value decreased moderately after nanovesicle immobilization, the *I–V* relationship remained linear. These results indicate that hormone sensors can maintain reliable electrical contacts, and that interaction between target hormones and nanovesicles can be detected by observing changes in current. In addition, the immobilization of the nanovesicle was indirectly proven through changes in the *I–V* values. Figure 3d shows the output curves of the hormone sensor in various *V*_*g*_ ranges from (0.1 to −0.7) V. The drain-source current (*I*_*sd*_) increases negatively according to the negatively increasing *V*_*g*_. Also, Fig. 3e displays the *I*_sd_–*V*_g_ transfer curve at a constant drain voltage (a constant value of *V*_sd_ = –100 mV). *V*_g_ was applied from –1 to 1.5 V at a sweep rate of –50 mV. *I*_sd_ increased as *V*_g_ was reduced from 0.35 to –1.0 V, which corresponds to the output characteristics seen in Fig. 3d. The result shows that the FET-graphene sensor maintains p-type behavior under our experimental conditions. Moreover, the linear properties (ohmic contact) of the *I–V* curves were maintained under the various *V*_*g*_, implying that the electrostatic gating effect can be the major influence leading to the current changes in the hormone sensor.

### Real-time responses of hormone sensors

To evaluate the sensing properties of the hormone receptor-carrying nanovesicle-based FET type hormone sensors, phosphate-buffered solution (PBS) containing 2 mM Ca^2+^ was used, which helped maintain effective gate regulation. The real-time responses of the liquid-ion gated FET sensors were measured at the p-type region. Graphene-based sensors presented ambipolar (p-type or n-type) properties, but exhibited a more sensitive and stable performance in the p-type region, due to the adsorption of oxygen from water or air. The main function of hormone receptor-carrying nanovesicles is to bind to hormone molecules, and signals derived from binding events activated the cAMP pathway of nanovesicles^42^. Activation of the Ca^2+^ channel induces influx of Ca^2+^ into the nanovesicles, which accumulates the potential of the nanovesicles immobilized on the graphene-FET. The size of the nanovesicles on the surface of the graphene is 100 nm. For this size, a screening effect dominates while charge detection is severely hampered^43^. However, Ca^2+^ ions flew into the nanovesicles after ligand binding. The accumulated Ca^2+^ caused a positive gate effect on the surface of the hormone sensor. The current decreased when the gate voltage was positively increased, as shown in Fig. 3d. Therefore, we can say the positive gate effect reduces the current of the p-type graphene -FET. This type of current decrease was demonstrated with a shift of the Dirac point of our group research^27^. In addition, our previous study showed that FET sensors could not detect signals when using buffer without Ca^2+^ or when Ca^2+^ channel blocker (MgCl_2_) was added to normal sensing buffer^31^. Moreover, the signal tended to increase when the concentration of Ca^2+^ in the buffer was increased^44^. These results support the conclusion that the signal change is caused by Ca^2+^ influx in the nanovesicles.

**Figure 4:**
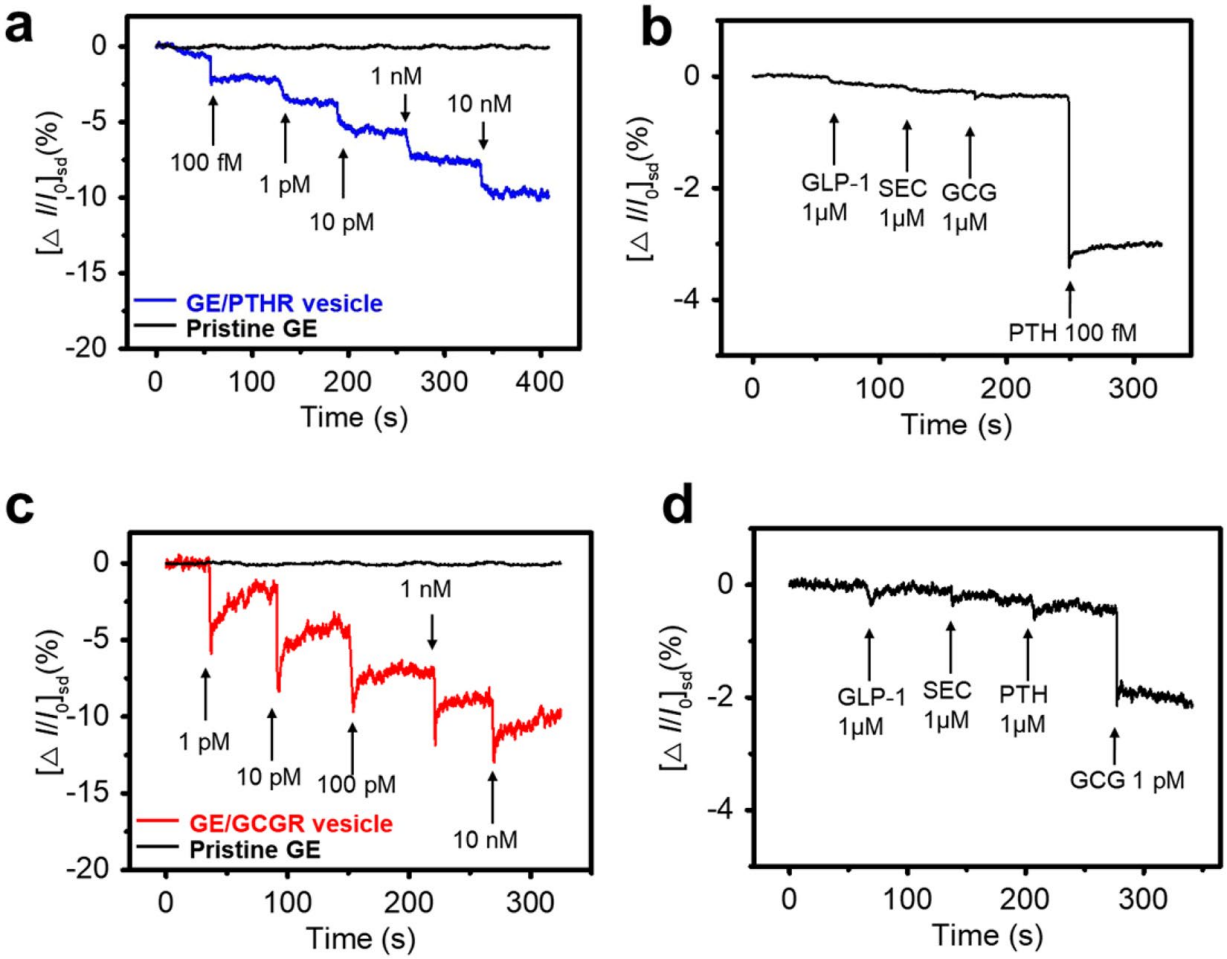
Real-time responses of the hormone sensors. (**a**) Real-time response of a PTH sensor with various concentrations of PTH (100 fM to 10 nM). (**b**) Selective response of a PTH sensor towards target hormone (PTH, 100 fM) and 1 µM of non-target hormones (glucagon-like peptide-1 (GLP-1), secretin (SEC) and GCG). (**c**) Real-time electrical measurements of GCG sensor with various concentrations of GCG (1 pM to 10 nM). (**d**) Selective property of a GCG sensor towards target hormone (GCG, 1 pM) and µM of non-target hormones.

Figure 4a,c show the real-time responses of the PTH sensor (Fig. 4a) and GCG sensor (Fig. 4c) observed by monitoring the *I*_sd_ after loading various concentrations of PTH and GCG. Negatively recorded currents can be explained by the reduction of the major carrier (hole) by the Ca^2+^ induction accumulated in the graphene. The specific binding of hormone to receptors on the nanovesicle promotes the activity of the calcium ion channel, allowing positive charges to be generated in the liquid ion gate dielectric near the graphene surface. As a result, carriers in the graphene channel, which are charged positively, decreased, resulting in a negative increase in current. From this detection mechanism, the nanovesicle-based hormone sensors have very sensitive response, but there is no significant signal from the pristine GE as control experiment. The minimum detection levels (MDL) of the PTH and GCG sensors were 100 fM and 1 pM, respectively, using the PTHR-carrying nanovesicles and the GCGR-carrying nanovesicles. The sensing signal immediately decreased upon the addition of PTH or GCG, indicating that the hormone sensor featured rapid response time of less than 1 second. The instantaneous current changes were monitored over a wide range of PTH/GCG concentrations (100 fM to 10 nM/1 pm to 10 nM). When the hormone sensor was interacted with higher concentrations of the hormones, the *I*_*sd*_ value gradually decreased.

Figure 4b,d show highly selective responses of the hormone sensors to other peptide hormones. With the addition of non-target hormone molecules, including glucagon-like peptide-1 (GLP-1), secretin (SEC) and PTH or GCG, no significant change in *I*_*sd*_ was monitored, but the current changes were clearly observed by the injection of PTH 100 fM or GCG 1 pM. These results indicate that the hormone sensors exhibit high selectivity, because hormone receptors on the nanovesicles have high specificity for the target ligand hormone.

## Discussion

In summary, two kinds of liquid-ion gated FET type hormone sensors were successfully demonstrated using graphene-FET and human hormone receptor-carrying nanovesicles. The electric field induction responses from the nanovesicle-based hormone sensors showed high detection performance at unprecedented low concentrations (ca. 100 fM of PTH and 1 pM of GCG). In addition, hormone sensors based on hormone receptor-carrying nanovesicles were able to distinguish hormones from non-target molecules. All reactions were detected within 1 second, and real-time detection was possible. This method seems to be able to measure hormones in a much faster and more accurate way than conventional peptide hormone detection methods. Therefore, these FET-type hormone sensors using human hormone receptor-carrying nanovesicles can be used as transducers for high-performance sensor applications, leading to a rapid and accurate methodology for the diagnosis and management of diseases.

## Methods

### Construction of hormone receptor-carrying nanovesicles

HEK-293 cells were cultured in Dulbecco’s modified Eagle medium (DMEM, Welgene, Republic of Korea), supplemented with 10% fetal bovine serum and 1% penicillin-streptomycin (Gibco, USA) at 37 °C under 5% CO_2_. HEK-293 cells were transfected with pDsRed-N1 containing human PTHR and GCGR, using Lipofectamine3000 (Invitrogen, USA), according to the manufacturer’s instructions. After the transfection, the expression of PTHR and GCGR was confirmed by fluorescence microscopy, and then cells were cultured for 1 day, and transferred to the media containing G-418 (1 mg/ml) for selection. After 1 week, G-418 resistant cell colonies were separately picked up, and cultured in fresh medium containing G-418. The cells stably expressing hormone receptors were suspended in serum-free DMEM containing cytochalasin B (20 μg/ml, Sigma, USA), and incubated at 37 °C with 300 rpm agitation for 20 min. Nanovesicles were separated from cells and cell debris by centrifugation (1,000 g, 5 min for cells, 2,000 g, 20 min for cell debris) in an Eppendorf tube, and collected by centrifugation at 12,000 g for 30 min. The nanovesicles were finally resuspended in Dulbecco’s phosphate buffered saline (DPBS, Gibco, USA). The produced nanovesicles were used immediately, or stored at –80 °C for subsequent experiments.

### Intracellular calcium assay

For calcium signalling analysis, stable cell lines expressing PTHR and GCGR were incubated in Ringer’s solution (140 mM NaCl, 1 mM MgCl_2_, 1.8 mM CaCl_2_, 5 mM KCl, 5 mM glucose, and 10 mM HEPES (pH 7.4)) containing 10 mM Fura-2/AM (Invitrogen, USA) for 30 min at 37 °C. After incubation, the cells were washed several times with Ringer’s solution without Fura-2/AM, and the cleavage of the AM ester group was followed by incubation for 1 h at 37 °C. Using a spectrofluorophotometer (Tecan, Switzerland), the fluorescence signal upon the addition of hormone was measured at 510 nm by dual excitation at (340 and 380) nm. For calcium analysis of nanovesicles, nanovesicles carrying PTHR and GCGR were generated from Fura2-AM loaded cells. Next, 96-wells plates were treated with poly-D-lysine, and the nanovesicle solutions were then immobilized on the plates at 37 °C for 2 h. The calcium signals upon the addition of hormones were measured by the same processor of calcium signalling analysis.

### Fabrication of graphene electrode

Single-layer graphene was fabricated using a chemical vapor deposition (CVD) method, and prepared on Cu foil (25 μm-thick). The size of the graphene channel was about 50 µm × 50 µm. The fabrication of GEs method is the same as previous study^45^.

### Immobilization of hormone receptor-carrying nanovesicles on GE

The above-prepared GEs were dipped in 1 mM 1-pyrenebutanoic acid N-hydroxysuccinimidyl ester (PSE) solution in methanol for 30 min at ambient temperature. To remove excess PSE, the GEs were washed three times with pure methanol. The PSE was introduced onto the graphene through π–π interaction between the surface of the graphene and the pyrene groups of the PSE. To build the nanovesicle-based hormone sensor, a 5 μl droplet of nanovesicle solution (1 mg/mL of total protein concentration) was used for the immobilization and placed on PSE treated GEs for 3 h. The proteins of the nanovesicle interacted with the succinimidyl groups of the PSE, resulting in chemically stable peptide bonds. Finally, the nanovesicle-immobilized GEs were rinsed with distilled water.

### Instrumentation

Glass chamber (200 μl Volume) was designed and placed on GE and filled with pH 7.4 DPBS containing 2 mM of CaCl_2_. The current change was observed and normalized as Δ*I/I*_0_ = (*I* − *I*_0_*)/I*_0_. Where *I*_0_ is the initial current and *I* is the measured real-time current.
